# PET-RAFT Polymerization Catalyzed by Small Organic Molecule under Green Light Irradiation

**DOI:** 10.3390/polym11050892

**Published:** 2019-05-15

**Authors:** Huazhen Tao, Lei Xia, Guang Chen, Tianyou Zeng, Xuan Nie, Ze Zhang, Yezi You

**Affiliations:** Hefei National Laboratory for Physical Sciences at the Microscale, CAS Key Laboratory of Soft Matter Chemistry, Department of Polymer Science and Engineering, University of Science and Technology of China, Hefei 230026, China; hzhtao@mail.ustc.edu.cn (H.T.); lxia@mail.ustc.edu.cn (L.X.); cg1995@mail.ustc.edu.cn (G.C.); ztyou@mail.ustc.edu.cn (T.Z.); niexuan@mail.ustc.edu.cn (X.N.)

**Keywords:** organic photoredox catalyst, green light, PET-RAFT polymerization

## Abstract

Photocatalyzed polymerization using organic molecules as catalysts has attracted broad interest because of its easy operation in ambient environments and low toxicity compared with metallic catalysts. In this work, we reported that 4,7-di(thiophen-2-yl)benzo[c][1,2,5]thiadiazole (DTBT) can act as an efficient photoredox catalyst for photoinduced electron transfer-reversible addition-fragmentation chain transfer (PET-RAFT) polymerization under green light irradiation. Well-defined (co)polymers can be obtained using this technique without any additional additives like noble metals and electron donors or acceptors. The living characteristics of polymerization were verified by kinetic study and the narrow dispersity (*Đ*) of the produced polymer. Excellent chain-end fidelity was demonstrated through chain extension as well. In addition, this technique showed great potential for various RAFT agents and monomers including acrylates and acrylamides.

## 1. Introduction

Photo-polymerization has received great attention because of its easy operation, temporal control, ambient condition, and so on. It has unique advantages in the synthesis of complex polymers, in bio-systems, and in microelectronics. Generally, visible light-driven polymerization is favored for its environmental friendliness and non-invasiveness. However, because conventional monomers have almost no absorption in visible region, a visible light-driven polymerization system generally requires photocatalysts or photosensitizers to harvest energy from visible light to trigger polymerization.

In recent years, visible light-driven polymerization has been rapidly developed, as many efficient photocatalysts or photosensitizers have been found, including metallic and non-metallic materials [1,2]. Owing to long excited lifetimes (microsecond) and high reductive abilities, transition metal compounds like Ir(ppy)_3_ and Ru(bpy)_3_Cl_2_ have exhibited high capabilities in photocatalyzed polymerization. Boyer et al. [3] reported photoinduced electron transfer-reversible addition-fragmentation chain transfer (PET-RAFT) polymerization using Ir(ppy)_3_ as a photocatalyst, which can be performed even in the presence of air. In addition, as a useful tool, this technique played an important role in the synthesis of multi-block copolymers [4,5], various stereopolymers [6], copolymers with well-defined sequence structures and sophisticated microstructures [7], and discrete oligomers [8]. Hawker et al. reported that Ir(ppy)_3_ could be utilized in visible light-induced atom transfer radical polymerization of methacrylates [9], acrylates [10], and to fabricate a 3D polymer brush [11]. Besides, many other metallic materials [12,13,14,15,16,17,18,19] have been developed to achieve living/controlled polymerization under irradiation by visible light as well.

Nevertheless, concerns of metal contamination in products have impeded its application; developing metal-free photo-polymerization is favored. Therefore, some dyes [20], phenazine derivatives [21], phenothiazine derivatives [22], phenoxazine derivatives [23], and other organic photocatalysts [24,25,26,27,28,29,30,31,32] have been constructed for visible light-polymerization. Fors [33,34], Nicewicz [35], and Boydston [36] et al. found that pyrylium salts can drive the polymerization of vinyl ethers, 4-methoxystyrene, and norbornenes, through cationic pathways or ring-opening metathesis mechanisms. Yagci et al. [37,38,39,40,41,42,43,44] developed visible light-cationic and radical polymerizations using various non-metallic materials. Even so, the very limited category and quantity of organic photocatalysts have motivated researchers to exploit new organic catalysts.

**Scheme 1:**
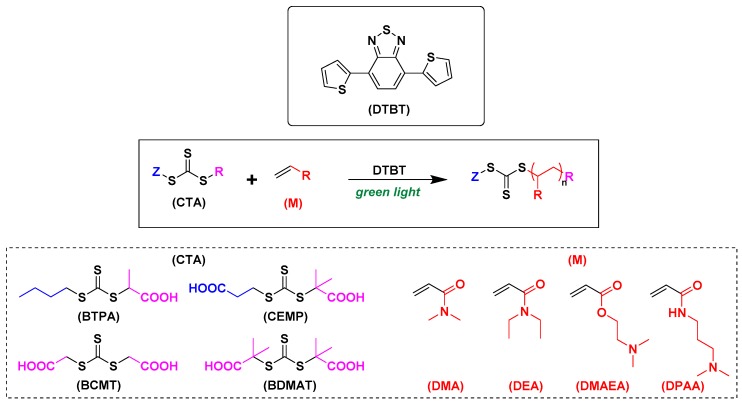
Diagram of photoredox catalyst for photoinduced electron transfer-reversible addition-fragmentation chain transfer (PET-RAFT), chemical structure of 4,7-di(thiophen-2-yl)benzo[c][1,2,5]thiadiazole (DTBT), and RAFT agents and monomers.

Small organic molecules with donor–acceptor–donor (D–A–D)-type π-conjugated structures play a significant role in organic photovoltaic devices because of efficient light-generated electron transfer processes [45]. With a high efficiency of charge separation and a long exciton lifetime, some of these organic molecules have great potential in the field of photocatalyzed polymerization. But few successful examples have been published [46,47]. In this study, we found an organic molecule comprising the thiophene as a donor unit (D), benzothiadiazole as an acceptor unit (A), and 4,7-di(thiophen-2-yl)benzo[c][1,2,5]thiadiazole (DTBT) (Scheme 1), which can catalyze PET-RAFT polymerization for synthesis of well-defined (co)polymers with low molecular weight distribution and high end-group fidelity of product under green light. In addition, this system has great compatibility in a variety of RAFT agents and monomers.

## 2. Materials and Methods

### 2.1. Materials

1-Butanethiol (Energy Chemical, Shanghai, China, 98%), 2-bromopropionic acid (Energy chemical, Shanghai, China, 99%), tetrabutylammonium hydrogen sulfate (Aladdin, Shanghai, China, 99%), 2,2’-(thiocarbonyldithio)diacetic acid, *N,N*-dimethyl acrylamide (DMA) (Aldrich, St. Louis, MO, USA, 99%), *N*,*N*-dimethylaminoethyl acrylate (DMAEA) (Energy chemical, Shanghai, China, 98%), *N,N*-Diethylacrylamide (DEA) (Aladdin, Shanghai, China, 99%), *N*-(3-(dimethylamino)propyl)acrylamide (DPAA) (Energy Chemical, Shanghai, China, 98%), 3-mercaptopropionic acid (Aladdin, Shanghai, China, 99%), 2-Bromoisobutyric acid (Energy chemical, Shanghai, China, 98%), 4,7-Di(thiophen-2-yl)benzo[c][1,2,5]thiadiazole (DTBT as photoredox catalyst in this study) (ARK, Chicago, IL, USA, >98.0%), bis(carboxymethyl) trithiocarbonate, CAS: 6326-83-6) (BCMT) (Energy Chemical, Shanghai, China, 98%), *N, N*-dimethylformamide (DMF) (Aladdin, Shanghai, China, 99.8%) were purchased from chemical company, respectively. Acetone, hydrochloric acid, carbon disulfide, chloroform, DCM, hexane, ether, K_3_PO_4_·3H_2_O, NaCl, NaOH, D_2_O, CDCl_3_, anhydrous Na_2_SO_4_ were obtained from Shanghai Chemical Reagent Co., Shanghai, China.

### 2.2. Characterization

All chemical structures were measured by a 400 MHz nuclear magnetic resonance (NMR) spectrometer (Bruker, Karlsruhe, Germany). The number-average molar mass (*M*_n,GPC_) and *Đ* were determined by a gel permeation chromatography (Waters, Milford, MA, USA) using DMF (1.0 mL/min) as the eluent with polystyrene standards. The absorption spectrum was measured by UV-Visible Spectroscopy (Sahimadzu, Kyoto, Japan).

### 2.3. Polymerizations

The polymerizations were conducted in a sealed glass tube, in a typical experiment, a 3 mL glass vial charged with monomer, chain transfer agent (CTA), catalyst and DMF as solvent. The mixture was deoxygenated by three freeze-pump-thaw cycles and sealed under vacuum. Then, it were irradiated under LED light at ambient temperature. Monomer conversions were measured by ^1^H-NMR, the final polymers were obtained by precipitation and analyzed by ^1^H NMR and GPC.

## 3. Results and Discussion

**Figure 1:**
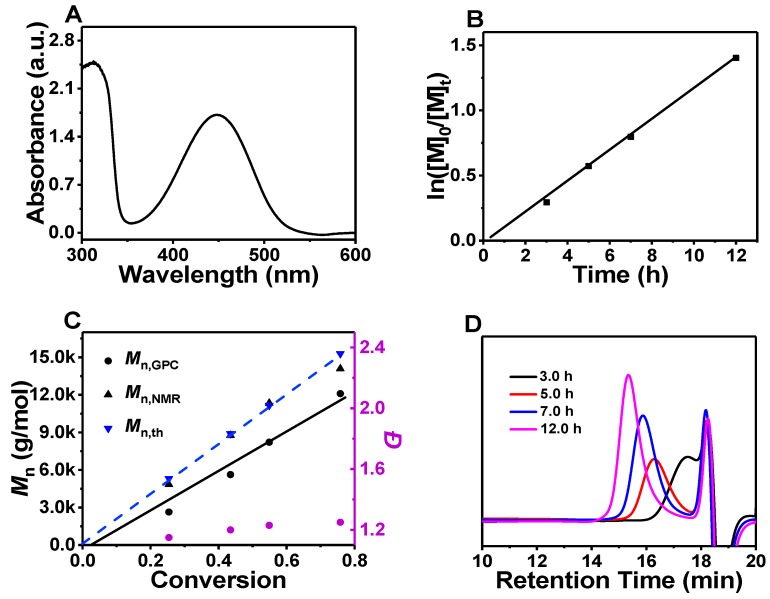
(**A**) The absorption spectrum of DTBT. Kinetic study of the PET-RAFT polymerization. Reaction conditions: [DMA]_0_:[CEMP]_0_:[DBTB]_0_ = 200:1:0.1, [DMA]_0_ = 3.0 M, DMF as solvent, the reactions were performed at room temperature under green LED. (**B**) The plot of ln([M]_0_/[M]_t_) as a function of reaction time. (**C**) The relationship of *M*_n,GPC_, *M*_n,NMR_, *M*_n,th_, and *Đ* versus monomer conversion. (**D**) GPC traces at different times of irradiation.

According to the reported PET-RAFT polymerization, the redox property and lifetime of the excited state of the photocatalyst are important [20]. DTBT is a small organic molecule semiconductor. It has a broad absorption area in visible light, even to 550 nm (Figure 1A), and can be excited by blue or green light. It has a low reduction potential value of –1.15 V and a long-living photogenerated exciton, [48] which is comparable with the mostly used transition metal photocatalyst (Ru(III)/Ru(II)* = –0.8 V) [12]. Therefore, we can envision that DTBT will be a promising photocatalyst for PET-RAFT polymerization.

In order to verify whether DTBT was effective for PET-RAFT polymerization, we chose *N, N*-dimethylacrylamide (DMA) as the model monomer, 2-((((2-carboxyethyl)thio)carbonothioyl)thio)-2-methylpropanoic acid (CEMP) (Figure S1) as the RAFT agent, *N, N*-dimethylformamide (DMF) as the solvent, and a blue (λ_max_ = 460 nm) or green (λ_max_ = 530 nm) LED as the light source. When in the presence of DTBT, poly-(*N,N*-dimethylacrylamide) (PDMA) with an *M*_n_ of 11600 Da and a narrow dispersity (*Đ* = 1.23) formed after 12.0 h irradiation (Table 1, Entry 1, Figure S2), and higher conversion would be reached if we increased the amount of catalyst (Table 1, Entry 2-3, Figure S3). In ^1^H-NMR spectroscopy of the formed PDMA, the signal peak at 0.8 ppm was assigned to the proton of –CH_3_ from the RAFT agent of CEMP (Figure S2). Also, several control experiments were set to prove the roles of light, RAFT agent, and photocatalyst. As shown in Table 1, when we operated the experiment in dark conditions, no monomer consumption was detected over 12.0 h, indicating that polymerization was triggered by light. If we conducted polymerization under irradiation by green light without DTBT, no polymer was obtained because of very low monomer conversion (2.0%). But if blue light was used instead of green light, some polymers were obtained, because CEMP had an absorption in the blue region and a maximal absorption peak at 441 nm [49,50]. CEMP can be excited into the excitation state through the n→π* forbidden transition of the C=S group under blue light irradiation, then generate radicals, which could initiate polymerization [51,52,53]. Furthermore, a control experiment without the RAFT agent was also carried out, but no polymerization occurred either. All these results indicated that DTBT can act as a photocatalyst for PET-RAFT polymerization, and the trithiocarbonate molecule acted as both initiator and chain transfer agent.

**Scheme 2:**
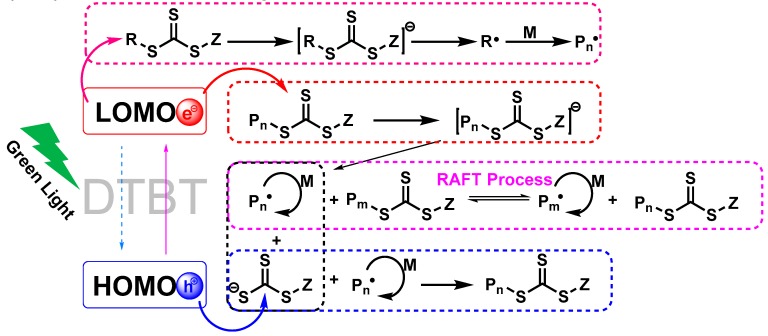
Outline of PET-RAFT polymerization in this study.

Based on the above study, we proposed the polymerization procedure in Scheme 2. DTBT generated conduction-band electrons (e^−^) and valence-band holes (h^+^) under green light. The photo-generated electron (e^−^) had stronger reduction activity than the RAFT agent. Hence, the RAFT agent was deoxidized to produce a radical, which provided a radical source to initiate RAFT polymerization The thiocarbonylthio compound acted as both initiator and chain transfer agent, which was why DTBT became indispensable in polymerization. At the same time, the radical (P_n_•) may also be deactivated by holes (h^+^) to regenerate the thiocarbonylthio compound, and the whole catalytic cycle would be restarted again.

As shown in Figure 1, the first order kinetic plot of ln([M]_0_/[M]_t_) as a function of polymerization time (Figure 1B) as well as the linear relationship between *M*_n,GPC_ of polymer and monomer conversion (Figure 1C) were observed. The plot of *M*_n,NMR_ (Figure 1C and Figure S4) versus monomer conversion gave a linear relationship in good agreement with that of the *M*_n,th_ (calculated by NMR). It demonstrated that almost constant active radicals existed in this system, and DTBT had remarkable activity in PET-RAFT polymerization of acrylamide driven by low-energy green light in ambient conditions. Moreover, GPC curves showed a single peak of produced polymer with narrow dispersity (*Đ* < 1.30), and it shifted to a short retention time (high molecular weight) region along with an increased polymerization time.

**Figure 2:**
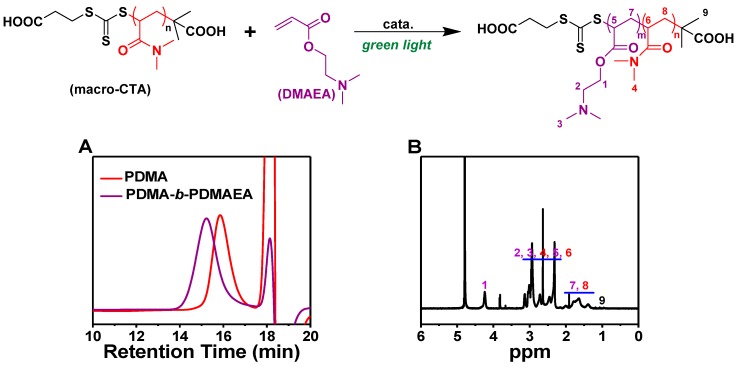
Chain extension of PDMA using PET-RAFT polymerization. Reaction conditions: [DMAEA]_0_:[macro-CTA]_0_:[DBTB]_0_ = 195:1:0.45, [DMAEA]_0_ = 1.3 M, DMF as solvent, the reactions were performed at room temperature under green LED light for 12.5 h. (**A**) GPC traces of PDMA and PDMA-*b*-PDMAEA. (**B**) ^1^H-NMR spectrum of PDMA-*b*-PDMAEA in D_2_O.

Chain extension is an available path to synthesize block copolymers to further attest fidelity of the polymeric chain, although the corresponding functional group has already been identified using ^1^H NMR spectroscopy. Chain extension of PDMA was performed using *N, N*-dimethylaminoethyl acrylate (DMAEA) as the second monomer. The shift of trace GPC product to the higher molecular weight side (Figure 2A) after the chain extension reaction and the signal peaks of protons of poly(*N,N*-dimethylaminoethyl acrylate) (PDMEA) and PDMA were all present (Figure 2B), verifying high end-group fidelity of PDMA.

**Figure 3:**
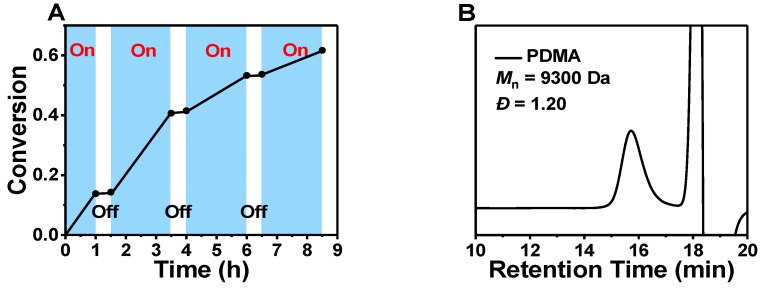
PET-RAFT polymerization of DMA in the presence (“On”) or in the absence (“Off”) of green light. Reaction conditions: [DMA]_0_:[CEMP]_0_:[DBTB]_0_ = 200:1:0.1, [DMA]_0_ = 3.0 M, DMF as solvent, the reactions were performed at RT under green LED. (**A**) Monomer conversion versus time. (**B**) GPC curve of final polymer.

To take full advantage of light stimulus, we performed “On” (in the presence of light) and “Off” (in the absence of light) experiments to investigate the temporal control of this DTBT-catalyzed PET-RAFT system. When the light was switched to the “On” state, polymerization occurred. When the light was in the “Off” state, no polymerization occurred based on ^1^H-NMR results (Figure 3A). The final product with a low *Đ* of 1.20 and an expected molecular weight in line with the trait of living/controlled polymerization was obtained (Figure 3B), which showed good ability of this system in the temporal control process.

In light of the versatility of PET-RAFT polymerization, it could be used in broad monomers and a variety of RAFT agents. Therefore, we envisioned that our DTBT-catalyzed PET-RAFT system had the same capabilities. As shown in Table 2, *S,S′*-bis(α,α′-dimethyl-α″-acetic acid)trithiocarbonate (BDMAT) (Figure S5), 2-(*n*-butyltrithiocarbonate)-propionic acid (BTPA) (Figure S6), and bis(carboxymethyl) trithiocarbonate (BCMT) were used as RAFT agents in this system instead of CEMP. As expected, all polymerizations (Figure S7–S9) were carried out successfully using these trithiocarbonates as chain transfer agents and initiators, and as a result, polymers with narrow dispersity and unimodal peaks in GPC curves were obtained.

Subsequently, some other monomers, such as *N*-(3-(dimethylamino)propyl)acrylamide (DPAA), *N, N*-dimethylaminoethyl acrylate (DMAEA), *N, N*-diethylacrylamide (DEA) and *N*-(3-(dimethylamino)propyl)acrylamide (DPAA), were applied in this system. Polymerizations of DPAA, DMAEA, and DEA were carried out under the same conditions as those of DMA. Polymerization of DEA resulted in a conversion of 85.9% with a low *Đ* of 1.31, which was similar to that of DMA because of their similar structures. Polymerization of DMAEA and DPAA had lower monomer conversions than DMA and DEA, but still produced polymers (Figure S10–S13) with low *Đ,* revealing the generality and compatibility of DTBT as a photocatalyst for the polymerization of these two monomers.

## 4. Conclusions

In conclusion, we developed a small organic molecule, DTBT, as photocatalyst for PET-RAFT polymerization. DTBT had noticeable activity in catalyzing PET-RAFT polymerization of common monomers (acrylamides and acrylates), and applying in several chain transfer agents without any help of sacrificial reagents or precious metal co-catalysts. Furthermore, this DTBT-catalyzed system showed excellent control over molecular weight, dispersity, and high group fidelity with successful chain extension.

## Figures and Tables

**Table 1:** Results of poly-(*N,N*-dimethylacrylamide) (PDMA) synthesized under different conditions. ^a^

Entry	[DMA]_0_:[CEMP]_0_:[DTBT]_0_	conv. (%) ^d^	*M*_n,th_^e^ (Da)	*M*_n,GPC_^f^ (Da)	*Đ* ^f^
1	200:1:0.1	78.9	15900	11600	1.23
2	200:1:0.2	87.5	17500	12700	1.25
3	200:1:0.02	50.0	10170	6200	1.32
4 ^b^	200:1:0.1	0	0	-	-
5	200:1:0	0	0	-	-
6 ^c^	200:1:0	18.0	2830	-	-
7	200:1:0.1	2.0	660	-	-

^a^ Reaction conditions: *N,N*-dimethylacrylamide [DMA]_0_ = 3.0 M, *N,N*-dimethylformamide (DMF) as solvent, the reactions were performed at room temperature under green LED light for 12.0 h. ^b^ In dark conditions. ^c^ Blue LED as light source. ^d^ Monomer conversion was determined by ^1^H-NMR spectroscopy. ^e^ *M*_n,th_ = [DMA]_0_/[2-((((2-carboxyethyl)thio)carbonothioyl)thio)-2-methylpropanoic acid (CEMP)]_0_ × MW^DMA^ × conv. + MW^CEMP^, where [DMA]_0_, [CEMP]_0_, MW^DMA^, conv., and MW^CEMP^ correspond to DMA and CEMP concentration, molar mass of DMA, monomer conversion, and molar mass of CEMP. ^f^ *M*_n,GPC_ and *Đ* were determined by GPC with PS standards.

**Table 2:** Results of polymers synthesized by PET-RAFT polymerization ^a^.

Entry	CTA	Monomer	conv. (%) ^b^	*M*_n,th_^c^ (Da)	*M*_n,GPC_^d^ (Da)	*Đ* ^d^
1	*S, S′*-bis(α, α′-dimethyl-α″-acetic acid)trithiocarbonate (BDMAT)	DMA	65.7	13300	7700	1.62
2	2-(n-butyltrithiocarbonate)-propionic acid (BTPA)	DMA	35.1	7190	2400	1.40
3	bis(carboxymethyl) trithiocarbonate (BCMT)	DMA	45.2	9180	7300	1.63
4	CEMP	DEA	85.9	21800	12040	1.31
5	CEMP	DMAEA	61.5	17900	25500	1.38
6	CEMP	DPAA	57.6	18200	6540	1.27

^a^ Reaction conditions: [DMA]_0_:[CEMP]_0_:[DBTB]_0_ = 200:1:0.1, [M]_0_ = 3.0 M, DMF as solvent, the reactions were performed at room temperture under green LED for 12.0 h. ^b^ Monomer conversion was determined by ^1^H NMR spectroscopy. ^c^ *M*_n,th_ = [DMA]_0_/[CEMP]_0_ × MW^DMA^ × conv. + MW^CEMP^, where [DMA]_0_, [CEMP]_0_, MW^DMA^, conv., and MW^CEMP^ correspond to DMA and CEMP concentration, molar mass of DMA, monomer conversion, and molar mass of CEMP. ^d^ *M*_n,GPC_ and *Đ* were determined by GPC with PS standards.

## Supplementary Materials

The following are available online at https://www.mdpi.com/2073-4360/11/5/892/s1. Figure S1: ^1^H-NMR spectrum of CEMP, Figure S2: ^1^H-NMR spectrum and GPC curve of PDMA (CEMP as RAFT agent), Figure S3: GPC curve of PDMA, Figure S4: ^1^H-NMR spectra of obtained PDMA in D_2_O at different reaction time (Figure 1C) (*M*_n,NMR_ = 3 × (I^1^)/(I^2^) × MW^DMA^ + MW^CEMP^)., Figure S5. ^1^H-NMR spectrum of BDMAT, Figure S6: ^1^H-NMR spectrum of BTPA, Figure S7: ^1^H-NMR spectrum and GPC curve of PDMA (BCMT as RAFT agent), Figure S8: ^1^H-NMR spectrum and PDMA curve of PDMA (BTPA as RAFT agent), Figure S9: ^1^H-NMR spectrum and GPC curve of PDMA (BDMAT as RAFT agent), Figure S10: ^1^H-NMR spectrum of PDMAEA, Figure S11: ^1^H-NMR spectrum of PDEA, Figure S12: ^1^H-NMR spectrum of PDPAA, Figure S13: GPC curves of PDEA, PDMAEA and PDPAA.
