# Supplementary material for: PET-RAFT Polymerization Catalyzed by Small Organic Molecule under Green Light Irradiation
Source: Polymers (Basel). 2019 May 15;11(5):892. doi: 10.3390/polym11050892 (PMC6572999; doi:10.3390/polym11050892)
Supplement: Supplementary file 1 [file polymers-11-00892-s001.pdf]

polymers

MDPI

Supporting Information

1. *Light source*

Blue LED light strip (5.76 W) and green LED light strip (6 W) bought from e-trade platform.

2. Synthesis of 2-((((2-carboxyethyl)thio)carbonothioyl)thio)-2-methylpropanoic acid (CEMP) [1]

In a 500 mL round-bottom-flask 3-mercaptopropionic acid (2.4 mL, 27.56 mmol, 1.1 eq.) was dissolved in a suspension of $K_3PO_4 \cdot 3H_2O$ (7.33 g, 27.56 mmol, 1.1 eq.) in acetone (160 mL) at ambient temperature. After stirring for 20 min, carbon disulfide (5.0 mL, 82.80 mmol, 3.3 eq.) was added and the color of solution turned to yellow. 2-Bromoisobutyric acid (2.09 g, 12.53 mmol, 1.0 eq.) was added after 20 min and the mixture was stirred overnight. 1.0 M HCl (400 mL) was added and the aqueous phase was extracted with DCM (300 mL). The combined organic extracts were washed with deionized H_2O, brine, dried over Na_2SO_4 and filtered. After evaporation of the solvent, the yellow solid was recrystallized two times from acetone (25 mL) to give the product as yellow crystals (2.02 g, 7.54 mmol, 27.4%).

3. Synthesis of 2-(n-butyltrithiocarbonate)-propionic acid (BTPA) [2]

Specifically, a 50% NaOH solution (3.28 g), 1-butanethiol (3.61 g, 4.3 mL), acetone (1.58 g, 2.0 mL) and water (6.0 mL) were added into three-neck flask under N_2 atmosphere and stirred for 30 min. The solution treated with carbon disulfide (2.7 mL, 3.42 g) to give a clear orange solution. It was stirred for 0.5 hour then cooled in an ice bath to an internal temperature below 10°C, 2-bromopropionic acid (6.27 g, 3.8 mL) was added slowly, followed by added with 3.28 g of 50% NaOH solution, stirred for 30 min again, the ice bath was removed and water (6 mL) was added, the mixture was stirred at room temperature for 20-24 hour. Then diluted with water (10 mL) and stirred and cooled in an ice bath for 20 min, while 10.0 M HCl (6 mL) was added at a rate which kept the temperature below 10 °C. A yellow oil separated, and stirring of the mixture was continued 1.0 hour at ice temperature until the oil solidified. The solid was collected by suction filtration, washed with water, and dried under high vacuum to a state of semi-dryness, which was recrystallized from hexane (10-12 mL) to give bright yellow microcrystals (5.52 g, 57.9%).

4. Synthesis of S, S′-bis(α, α′-dimethyl-α′′-acetic acid)trithiocarbonate (BDMAT) [3]

Carbon disulfide (13.77 g, 0.018 mol), chloroform (58.81 g, 0.45 mol), acetone (26.26 g, 0.45 mol), and tetrabutylammonium hydrogen sulfate (1.21 g, 3.6 mmol) were mixed with 12 m L of hexane in a 500 mL round bottom. Then NaOH solution (50%, 100.8 g) was added into the mixture over 1.0 h while maintaining the temperature below 25 °C. After the addition was complete the reaction was maintained at 22-25 °C for 10 h while being stirred with a magnetic stirrer. 90 mL H_2O was added to dissolve the yellow solids, followed by adding hydrochloric acid (12 mL) to acidify the aqueous solution and yield crystalline solids. After filtration and washing three times by H_2O, the crude compound was purified by recrystallization several times in acetone and hexane (4:1 v/v) to give the product as yellow crystals (2.45 g).

5. General procedure for kinetic study of photoinduced RAFT polymerization.

The polymerization was conducted in a glass tube with rubber. A 10 mL glass vessel charged with DMA (3.0 M), trithiocarbonate (CEMP), catalyst ($[DMA]_0$:$[CEMP]_0$:$[cata.]_0$ = 200:1:0.1) and DMF as solvent. The mixture was deoxygenated by three freeze-pump-thaw cycles, followed by sealed under Ar atmosphere. The sealed glass tube was irradiated by green LED light at ambient temperature. Aliquots were withdrawn by syringes from the reaction mixture at different time, polymer was obtained by precipitation and analyzed by ^{1}H NMR, GPC.

6. General procedure for "On-Off" expereiment of photoinduced RAFT polymerization.

The polymerization was conducted in a glass tube with rubber. A 10 mL glass vessel charged with DMA (3.0 M), trithiocarbonate (CEMP), catalyst ($[DMA]_0:[CEMP]_0:[cata.]_0$ = 200:1:0.1) and DMF as solvent. The mixture was deoxygenated by three freeze-pump-thaw cycles, followed by sealed under Ar atmosphere. The sealed glass tube was alternately irradiated by green LED light ("On" state) or set in dark environment ("Off" state) at ambient temperature. Aliquots were withdrawn by syringes from the reaction mixture at different time, final polymer was obtained by precipitation and analyzed by ^{1}H NMR, GPC.

7. General procedure for chain extension of photoinduced RAFT polymerization.

A typical experiment procedure of chain extension as follow: DMAEA (92.7 mg, 0.65 mmol), PDMA (macro-CTA, M_n = 11500 Da, $Đ$ = 1.22, 38.2 mg, 0.0033 mmol), catalyst (0.45 mg) and DMF (V_{tol} = 0.5 mL) were added into a glass tube. Subsequently, the mixture was degassed by three freeze-pump-thaw cycles. The tube was sealed under vacuum and irradiated by green LED light at ambient temperature for 12.5 h. After stop polymerization, Monomer conversion was measured in ^{1}H-NMR, the final polymer was obtained by precipitation and analyzed by ^{1}H NMR, GPC.

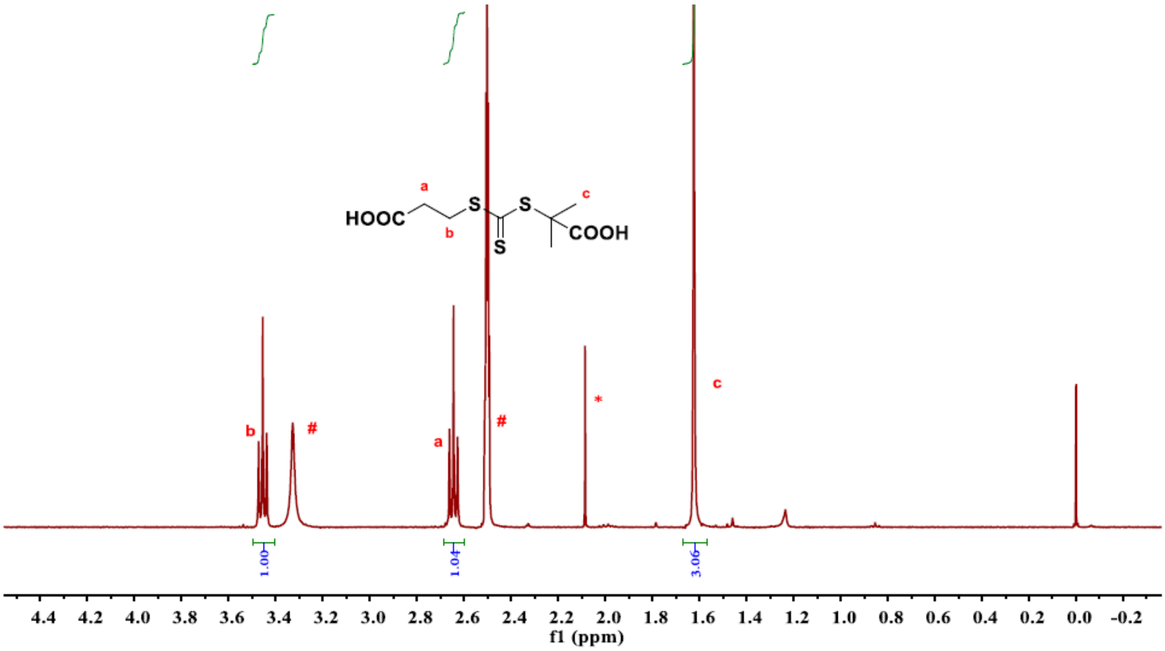

Figure S1. ^{1}H-NMR spectrum of CEMP.

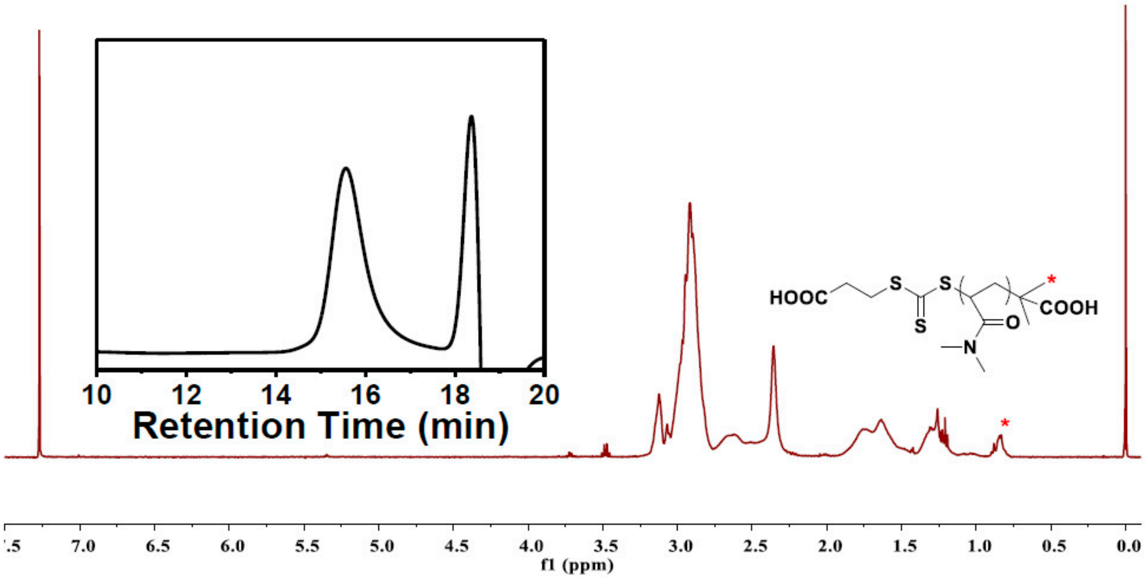

Figure S2. ^{1}H-NMR spectrum of and GPC curve of PDMA (CEMP as RAFT agent).

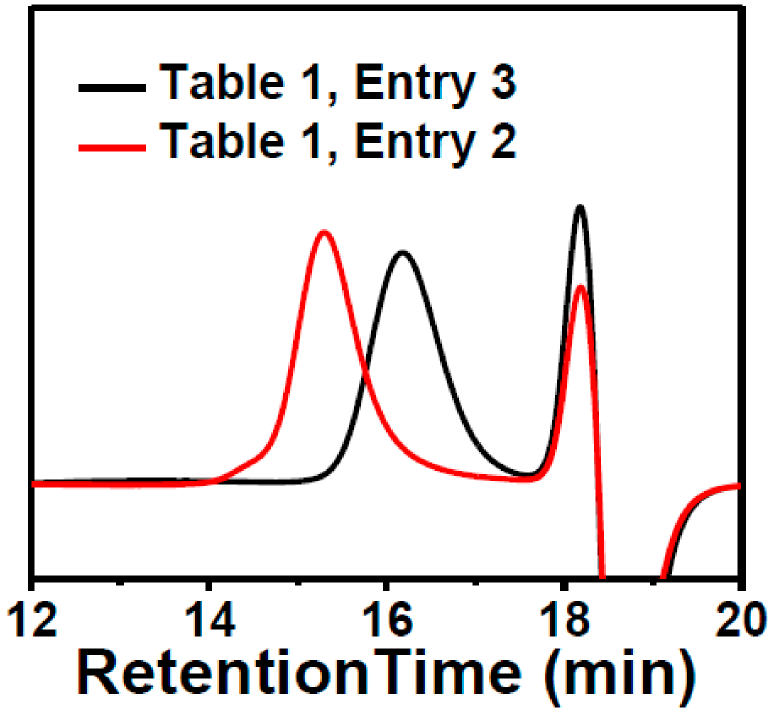

Figure S3. GPC curves of PDMA.

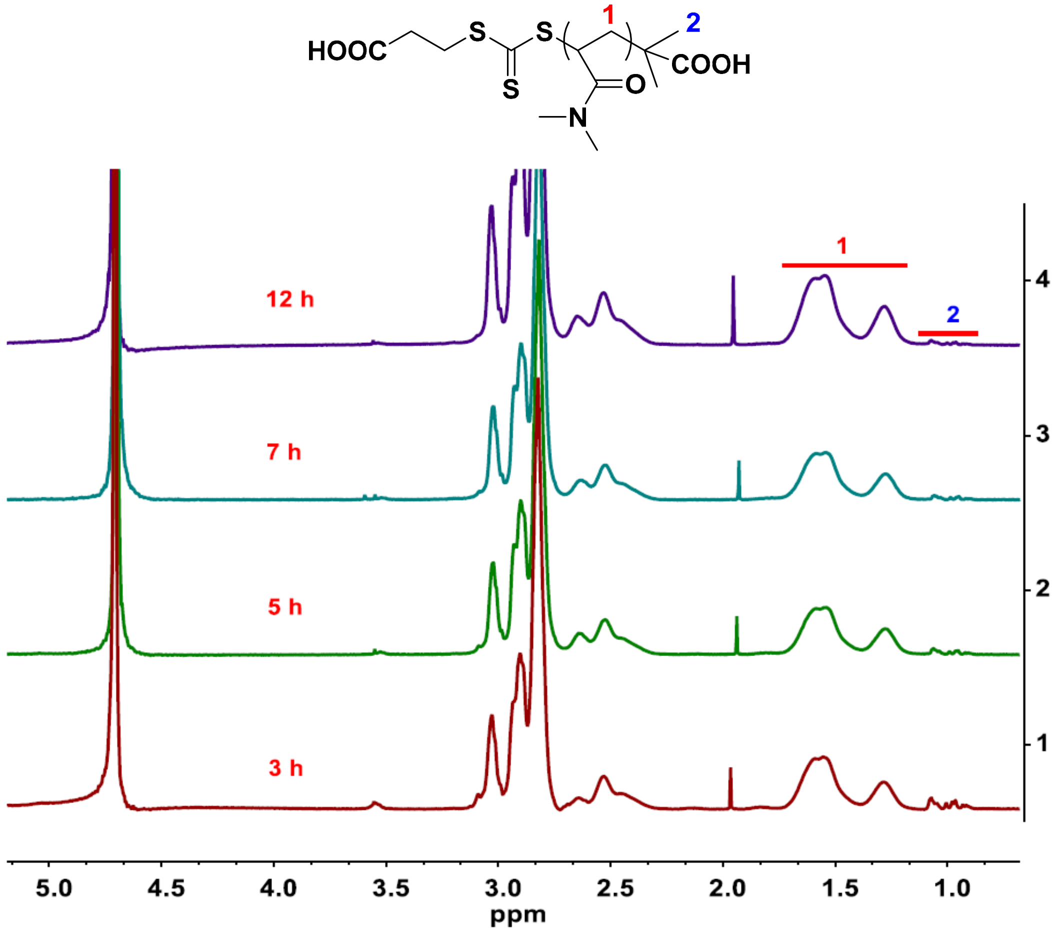

Figure S4. ^{1}H-NMR spectra of obtained PDMA in D_2O at different reaction time (Figure 1C) ($M_{n,NMR} = 3 \times (I^1)/(I^2) \times MW^{DMA} + MW^{CEMP}$).

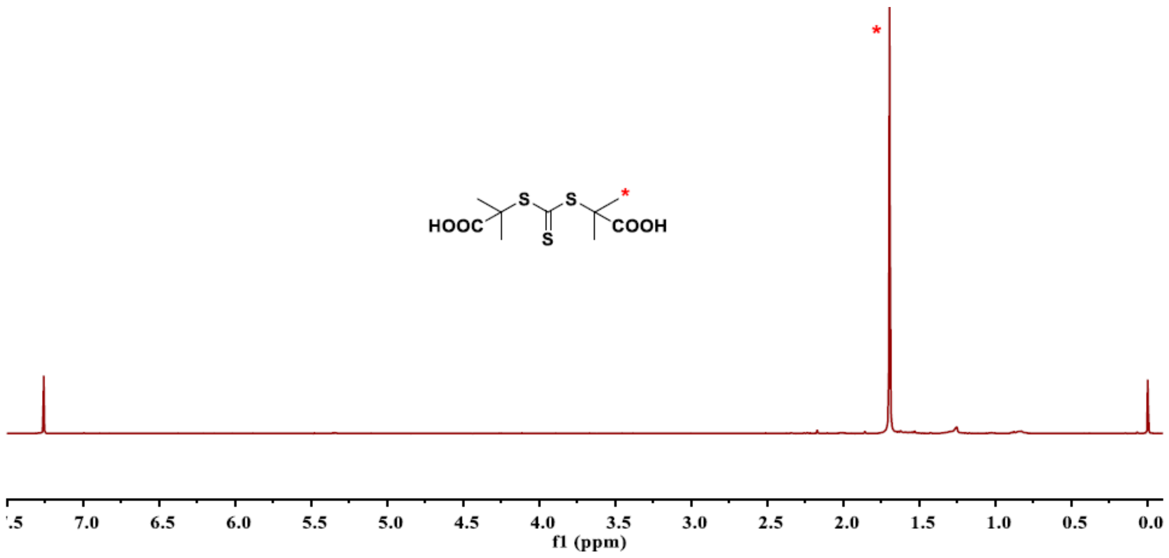

Figure S5. ^{1}H-NMR spectrum of BDMAT.

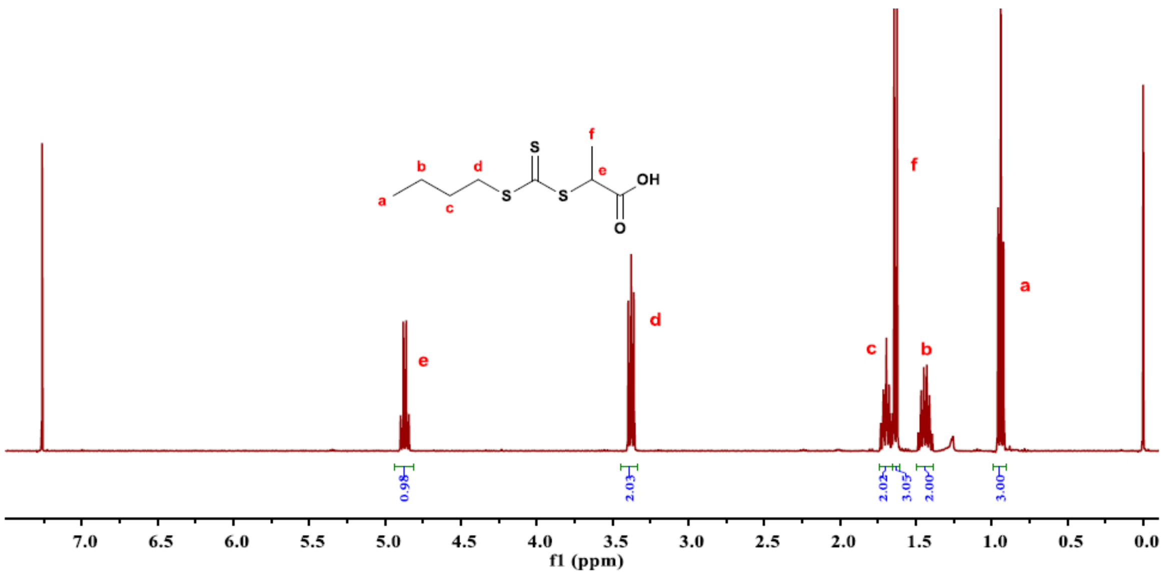

Figure S6. ^{1}H-NMR spectrum of BTPA.

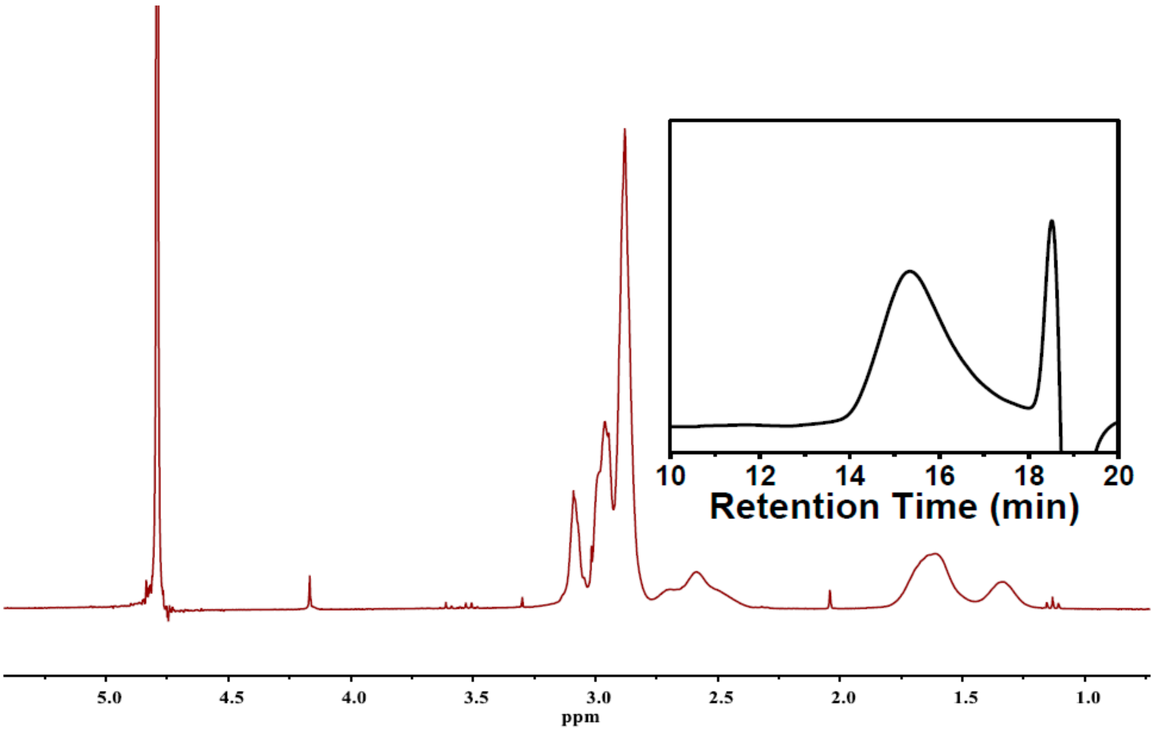

Figure S7. ^{1}H-NMR spectrum and GPC curve of PDMA (BCMT as RAFT agent).

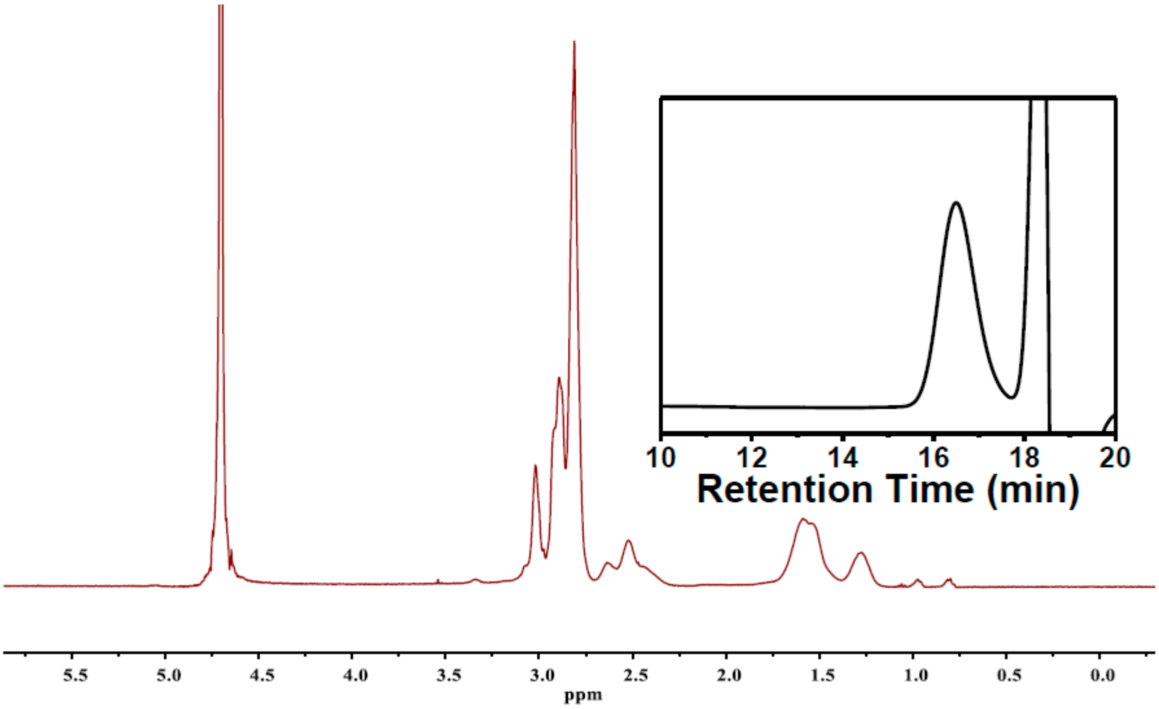

Figure S8. ^{1}H-NMR spectrum and GPC curve of PDMA (BTPA as RAFT agent).

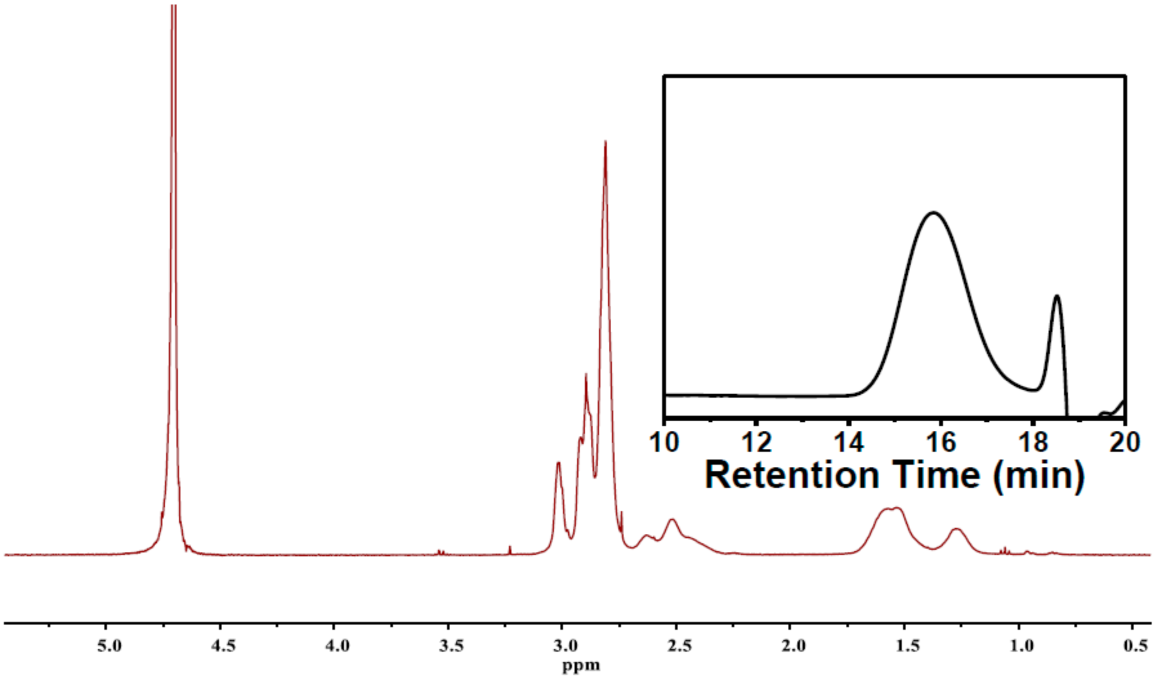

Figure S9. ^{1}H-NMR spectrum and GPC curve of PDMA (BDMAT as RAFT agent).

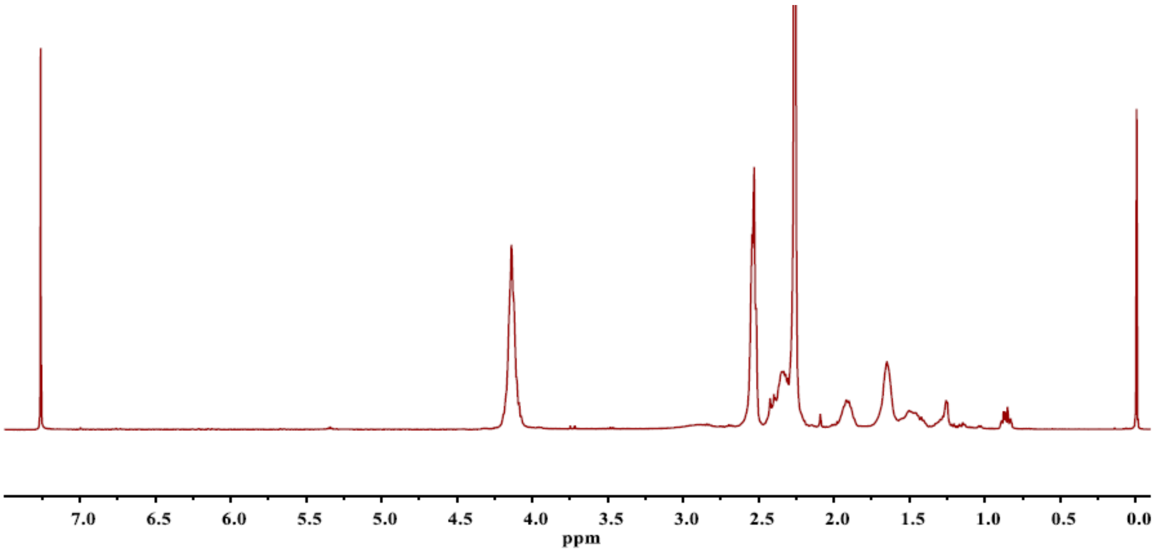

Figure S10. ^{1}H-NMR spectrum of PDMAEA.

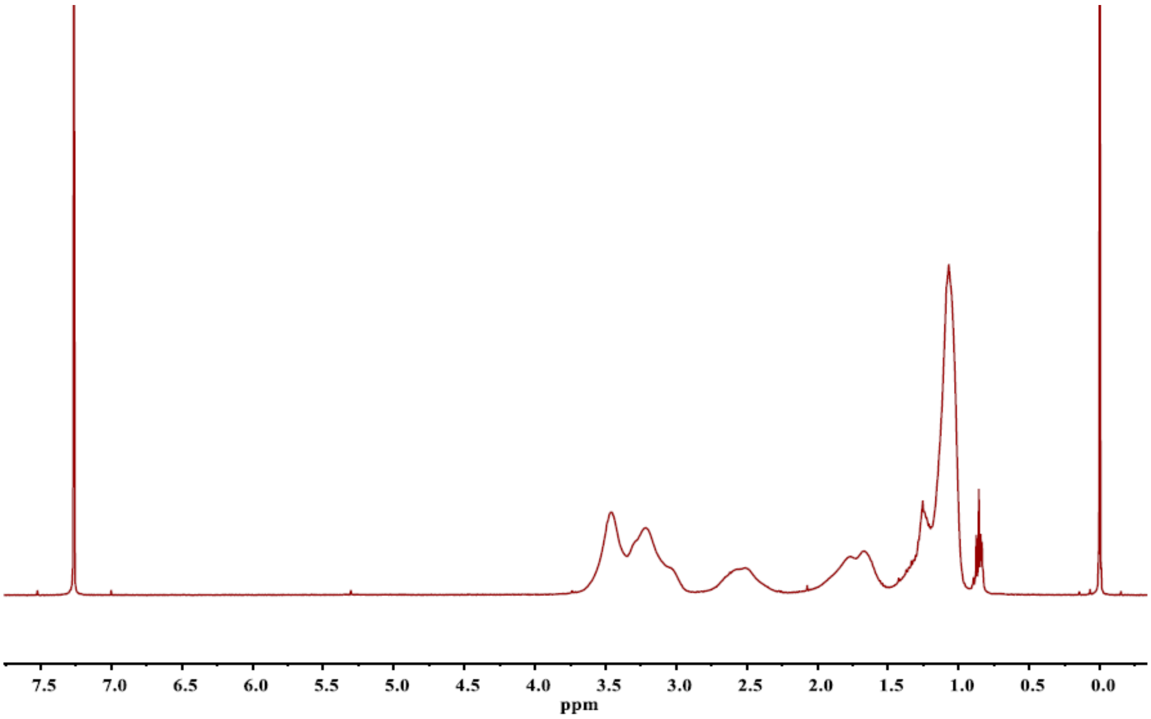

Figure S11. ^{1}H-NMR spectrum of PDEA.

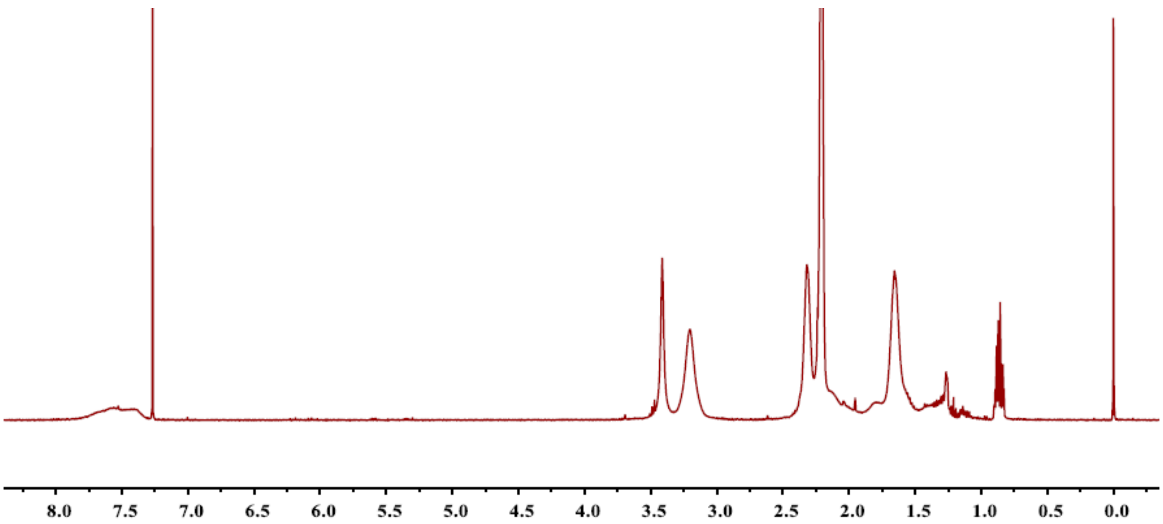

Figure S12. ^{1}H-NMR spectrum of PDPAA.

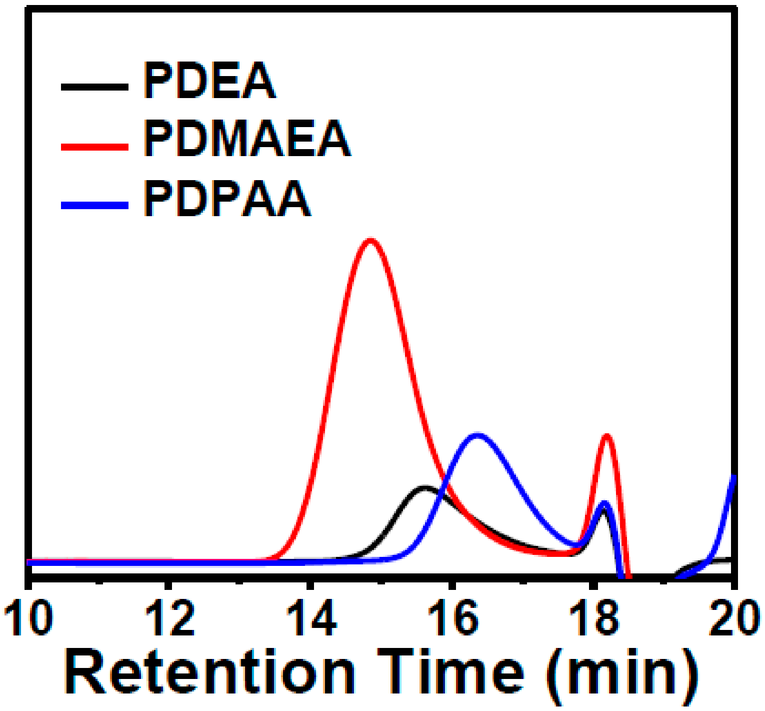

Figure S13. GPC curves of PDEA, PDMAEA and PDPAA.

References

1. Schmidt, B. V. K. J.; Hetzer, M.; Ritter, H.; Barner-Kowollik, C., UV Light and Temperature Responsive Supramolecular ABA Triblock Copolymers via Reversible Cyclodextrin Complexation. Macromolecules 2013, 46 (3), 1054-1065.
2. Ferguson, C. J.; Hughes, R. J.; Nguyen, D.; Pham, B. T. T.; Gilbert, R. G.; Serelis, A. K.; Such, C. H.; Hawkett, B. S., Ab initio emulsion polymerization by RAFT-controlled self-assembly. Macromolecules 2005, 38 (6), 2191-2204.
3. Wang, R.; McCormick, C. L.; Lowe, A. B., Synthesis and evaluation of new dicarboxylic acid functional trithiocarbonates: RAFT synthesis of telechelic poly(n-butyl acrylate)s. Macromolecules 2005, 38 (23), 9518-9525.

© 2017 by the authors. Submitted for possible open access publication under the terms and conditions of the Creative Commons Attribution (CC BY) license (http://creativecommons.org/licenses/by/4.0/).
